# Wavelength-Dependent DNA Damage Induced by Single Wavelengths of UVC Light (215 to 255 nm) in a Human Cornea Model

**DOI:** 10.21203/rs.3.rs-5129114/v1

**Published:** 2024-12-03

**Authors:** Manuela Buonanno, Raabia Hashmi, Camryn E Petersen, Zheng Tang, David Welch, Igor Shuryak, David J Brenner

**Affiliations:** Columbia University; Columbia University; Columbia University; Columbia University; Columbia University; Columbia University; Columbia University

## Abstract

Scientific bodies overseeing UV radiation protection recommend safety limits for exposure to ultraviolet light in the workplace based on published peer-reviewed data. To support this goal, a 3D model of the human cornea was used to assess the wavelength dependence of corneal damage induced by UVC light. In the first set of experiments the models were exposed with or without simulated tears; at each wavelength (215–255 nm) cells with DNA dimers and their distribution within the epithelium were measured. Simulated tears reduced the fraction of damaged cells to an extent dependent on the wavelength and tissue layer.

Another set of models were exposed without tears; yields of DNA-damaged cells and their distribution within the corneal epithelium were evaluated at each wavelength, together with other markers of cell and tissue integrity. Unlike relatively longer wavelengths, the range commonly referred to as far-UVC (215–235 nm) only induced dimers in the uppermost layers of the epithelium and did not result in lasting damage or halt proliferation of the germinative cells.

These results provide evidence for the recommended exposure limits for far-UVC wavelengths, which have been proposed as a practical technology to reduce the risk of transmission of airborne diseases in occupied locations.

## Introduction

Mitigating the risk of airborne disease transmission is crucial to public health goals worldwide. As seen during the COVID-19 pandemic, the rise of novel pathogens, for which treatments might not be immediately available, would require a lengthy and costly development of new vaccines.

An ideal approach to limit transmission of airborne diseases is direct inactivation of the airborne pathogens within a short time of their generation. Ultraviolet light has such ability; in fact, air disinfection with UVC fixtures primarily emitting energy at 254 nm has been demonstrated for a variety of airborne diseases including measles and tuberculosis [1–3]. However, exposure to conventional germicidal lamps emitting at 254 nm can cause adverse effects to skin and eye. Recently, a type of germicidal ultraviolet technology that utilizes shorter wavelengths in the 200–235 nm range, named far-UVC, has been proposed as a safer approach for continuous air disinfection in occupied indoor spaces.

Scientific agencies such as the American Conference of Governmental Industrial Hygienists (ACGIH^®^) and the International Commission on Non-Ionizing Radiation Protection (ICNIRP) regularly review published peer-reviewed data to formulate guidelines for the safe use of ultraviolet light in the workplace [4, 5]. For example, the ACGIH recommends UV exposure limits, referred to as Threshold Limit Values (TLVs), which represents conditions under which nearly all workers may be repeatedly exposed without adverse health effects to skin and eye [4]. To contribute towards improved recommendations of the UVC action spectrum and associated exposure limits, our previous studies have investigated the wavelength dependence of DNA photodamage induced in the whole epidermis and within the different epidermal layers of a realistic 3-dimensional (3D) model of human skin exposed to narrow bandwidth UVC generated by a monochromatic exposure system. Here we expanded those studies to a 3D model of the human cornea consisting of normal human corneal epithelial cells cultured to form a stratified (4–5 cell layers), squamous epithelium which closely parallels normal human corneal tissue [6].

Proteins absorb UVC radiation in peptide bonds at a rate that is wavelength dependent, with shorter wavelengths being more highly absorbed by proteins than longer wavelength [7–10]. In skin, this implies that the proteins in the uppermost layer of the exposed epidermis, *i.e.* the stratum corneum or the dead cell layer, absorb most of the incident far-UVC light so it does not reach and damage the DNA of the cells located deeper into the tissue [11–13]. Based on a similar biophysical rationale, we hypothesized that proteins in the human tear lm, which contains over 1500 proteins [14] together with water, electrolytes, and lipids [15], might afford protection to the exposed cornea [16]. To test our hypothesis, in the first set of experiments in this study we exposed 3D models of the human cornea with or without 10 μm of artificial tears (~ 15 mg/ml BSA) to 100 mJ/cm^2^ of narrow bandwidth UVC light ranging from 215 nm to 255 nm. Tissues were analyzed for induction of the pre-mutagenic cyclobutene pyrimidine dimers (CPD) and their distribution within the layers of the epithelium.

In the second set of experiments, the cornea model tissues were exposed without tears and, together with CPD, the tissues were examined for yields of the second most abundant DNA photodamage, 6 – 4 pyrimidine-pyrimidone photoproducts (6–4PP) and their distribution in the corneal epithelium. Correlation between UV-induced DNA dimers and their carcinogenic potential has been reported extensively [17–19]. Additionally, 24 h after exposure, cornea models were assessed for the presence of lasting γH2AX foci as a proxy of DNA double strand breaks.

Besides DNA damage, exposure to UVC can cause other types of adverse effects. To assess cell and tissue integrity, cornea models were evaluated for the percentage of proliferating corneal epithelial cells expressing the cell proliferation marker ki67, and for the induction of different modes of cell death including autophagy by measuring the expression of the autophagosome marker LC3B [20], and RIP1, a marker of necroptosis that is a caspase-independent type of cell death [21].

An intact cell-to-cell communication contributes to the maintenance of the human corneal epithelium homoeostasis. In the basal cell layer cells communicate with each other by allowing the intercellular passage of small molecules up to 1 kDa through gap junctions whose main associated protein is connexin 43 (cx43) [22]. Thus, gap junction intercellular communication is crucial for the regulation of cell growth, proliferation, and differentiation [23], therefore cornea models were evaluated for this as well.

The results from this study suggest that simulated tears reduced the fraction of CPD-positive cells to an extent that was dependent on the wavelength and the layer of the tissue. In human cornea models exposed without artificial tears, all far-UVC wavelengths tested (215 nm to 235 nm) induced DNA dimers but only in the uppermost superficial layers of the epithelium. Twenty-four hours after exposure, far-UVC wavelengths did not result in lasting DNA double strand breaks, halt proliferation of the germinative cells, induce cell death or impair cell-to-cell intercellular communication. In contrast, longer wavelengths induced these markers of cellular and tissue damage to some extent.

## Results

### Simulated tears reduced the induction of CPD in a human cornea model to an extent dependent on the wavelength and tissue layer.

**Figure 1:**
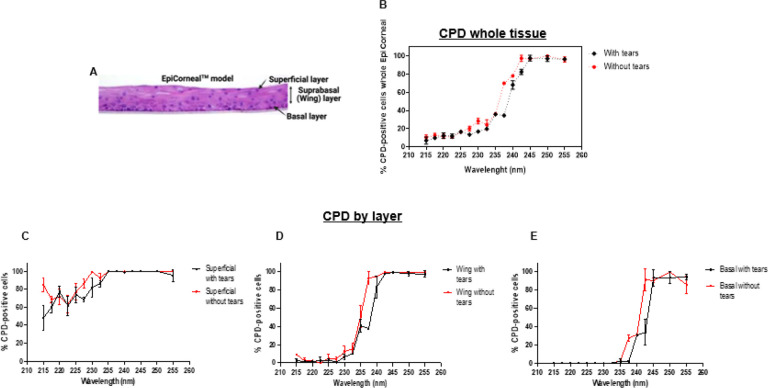
CPD in the whole corneal epithelium and in each layer of tissues exposed to different monochromatic UVC wavelengths with or without artificial tears. A) Cross-section of the model of the corneal epithelium with its layers used in this study. In tissues exposed with or without simulated tears (~15 mg/ml BSA) to 100 mJ/cm^2^ from different monochromatic UVC wavelengths we measured: B) the percentage of corneal cells showing CPD dimers and their distributions within the C) superficial, D) suprabasal or E) basal layer of the epithelium. Values represent the average ± SEM of cells exhibiting dimers measured in three to 15 randomly selected fields of view per samples (n=2, an average of approximately 1500 cells per sample were counted).

The two organs at major risk from UV exposure are skin and eye. Within the wavelengths we studied (215–255 nm) there is a range named far-UVC (200–235 nm) that has been shown to be highly effective at inactivating pathogens [24–26] while being minimally damaging to skin and eyes [16, 27, 28]. In skin, the proteins in the top layer of the epidermis (the stratum corneum or the dead cell layer) absorb most of the incident light [7, 12], thus far-UVC light cannot reach and damage the stem cells located in the epidermal basal layer [29, 30]. Like the stratum corneum for skin, the tear film that overlays the eye’s outer surface contains several proteins and it is predicted to afford similar protection to the exposed cornea [31]. To investigate whether this is the case, in the first set of experiments we exposed human cornea models to 100 mJ/cm^2^ generated by monochromatic UVC light overlaid or not by 10 μm of artificial tears (~ 15 mg/ml BSA) and compared the induction of CPD 30 minutes later. Figure 1 shows the percentage of cells with CPD as a function of wavelength in the whole corneal epithelium (Fig. 1A and 1B) and in each layer of the epithelium [superficial, suprabasal (Wing) and basal] exposed with or without tears (Fig. 1C–E).

To assess the wavelength above which the measured DNA damage was significantly greater than the zero-dose controls, Fisher’s exact test was used to compare the yield of CPD-positive cells per cell scored in each of the three corneal layers, with corresponding zero-dose controls. Raw p values and p values adjusted for multiple comparisons using the Bonferroni correction were recorded. The results are shown in Table 1 which, for each of the three corneal layers studied, shows the wavelengths above which the measured corneal DNA damage was significantly above control values. As can also be seen visually in Fig. 1E, in the key basal layer of the cornea, there was no significant increase in measured DNA damage at wavelengths below 240 nm (with tears) or 237.5 nm (without tears).

**Figure 2:**
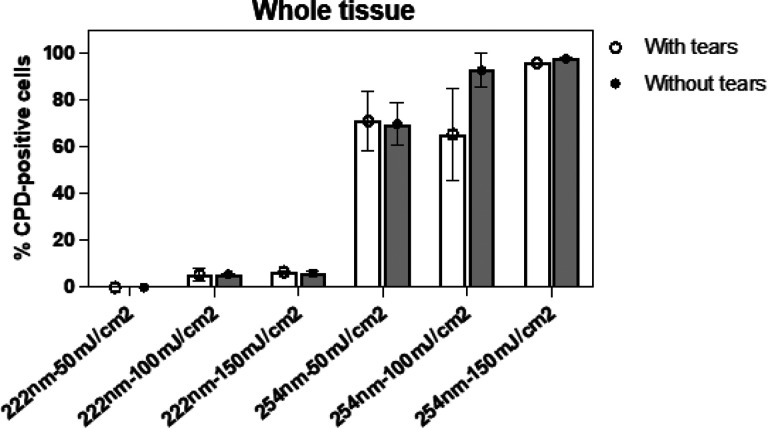
CPD in the whole corneal epithelium of tissues exposed to 222 nm or 254 nm light emitted by fixtures. Percentage of corneal cells showing CPD dimers in whole tissues exposed to 50, 100 or 150 mJ/cm^2^ generated by a filtered 222 nm krypton-chloride (KrCl) excimer lamp or by a low-pressure mercury lamp which principally emits at 254 nm. Values represent the average ± SD of cells exhibiting dimers measured in three to eight randomly selected fields of view per samples (n=2, an average of approximately 600 cells per sample were counted).

Results similar to those shown in Fig. 1B were obtained when we analyzed CPD induction in the whole epithelium of 3D cornea models exposed to 222 nm or 254 nm light generated by a filtered a krypton-chloride (KrCl) excimer lamp or a low-pressure mercury lamp, respectively (Fig. 2).

### Induction of DNA damage in human cornea models as function of UVC wavelength

**Figure 3:**
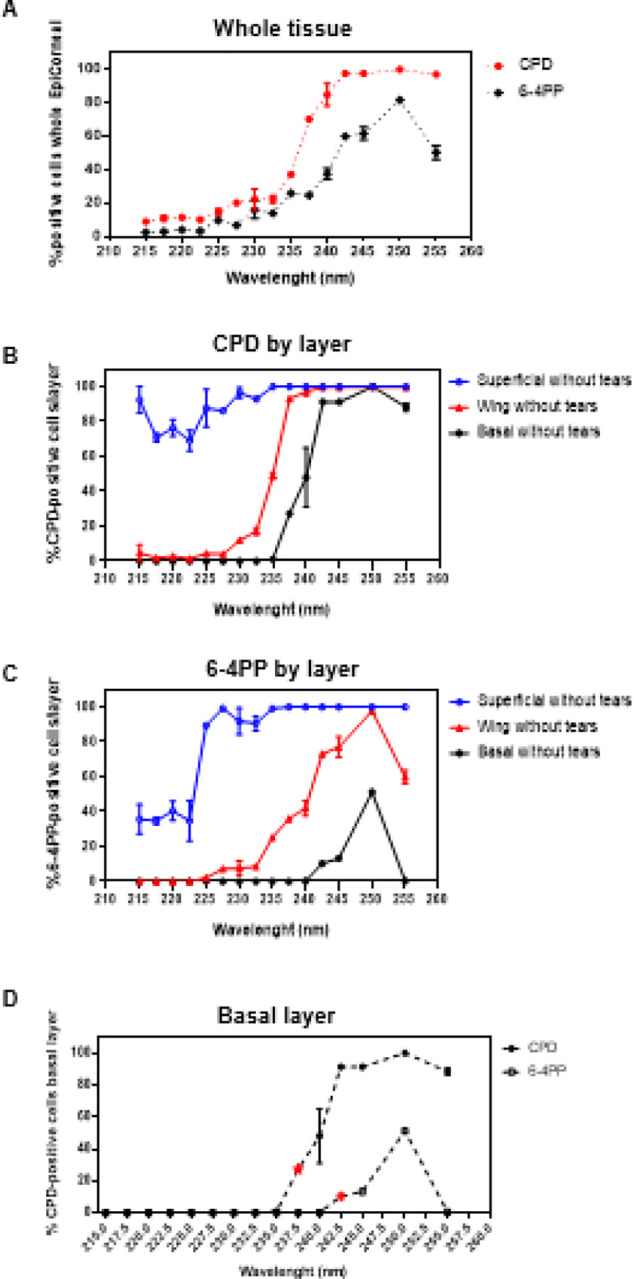
DNA dimers in the whole corneal epithelium and in each layer of tissues exposed to different monochromatic UVC wavelengths. A) Percentage of corneal cells showing CPD or 6–4 PP dimers in whole tissues as function of UVC wavelength (100 mJ/cm^2^). Distribution of B) CPD- or C) 6–4PP-positive cells in the different layers of the corneal epithelium. D) Percentage of CPD or 6–4PP in in the basal layer as function of UVC wavelength. Values represent the average ± SEM of cells exhibiting dimers measured in three to 15 randomly selected fields of view per samples (n=2, an average of approximately 1500 cells for each marker and sample were counted).

The second set of experiments was conducted with samples exposed to monochromatic UVC light without tears, which represent a more conservative model from the perspective of induced damage. In addition to CPD, 30 min after exposure we measured induction of the second most abundant pre-mutagenic DNA lesions, 6 – 4 photoproducts (6–4PP), in tissues exposed to 100 mJ/cm^2^ as function of wavelength in the total corneal epithelium (Fig. 3A) and in each epithelial layer (Fig. 3B and 3C).

Among the wavelengths studied, those in the far-UVC range (215–235 nm), induced CPD in approximately 20% of corneal cells. In the whole epithelium, the percentage of cells with CPD increased with longer wavelengths and plateaued starting at around 242.5 nm, reaching almost 100% in cells of tissues exposed to 250 nm (Fig. 3A). 6 – 4 PP (Fig. 3A) instead were induced on average in approximately 10% of cells of tissues exposed to wavelengths in the range of 215–227.5 nm; in the entire epithelium, the percentage of cells positive for 6–4PP reached a maximum of approximately 80% at 250 nm and then decreased to approximately 50% in cells of tissues exposed to 255 nm (Fig. 3A). The distribution of the CPD and 6–4PP in the different cell layers is shown in Fig. 3C and Fig. 3D, respectively. All wavelengths induced CPD in the uppermost layer of the exposed tissues; 6–4PP appeared in the superficial layer in an average of approximately 36% of cells of tissues exposed to 215–222.5 nm, reached approximately 90% at 225 nm, and then plateaued to almost 100% in cells of tissue exposed to higher wavelengths. In the suprabasal (Wing) layer, far-UVC wavelengths up to 232.5 nm induced CPD in less than 20% of the cells on average, while 6–4PP were measured in an average of 80% of cells for wavelengths in the 242.5–250 nm range. Like other stratified epithelia, cornea cells in the basal layer commit to terminal differentiation to replenish the superficial and suprabasal cells that sheds away during physiological replicative cell turnover [32, 33]. Thus, the most critical layer for the renewal of the corneal epithelium is the basal layer where stem cells and progenitors are located. In the basal layer, CPD and 6–4PP were higher than controls (p < 0.05) only in tissues exposed to 237.5 nm and 242.5 nm, respectively (Fig. 3D). While in tissues exposed to 242.5 nm and higher wavelengths almost 100% of basal cells had CPD, 6–4PP were detected in approximately 50% of the cells in tissues exposed to 250 nm; the percentage of 6–4PP-positive basal cells then decreased to close to zero in tissues exposed to 255 nm (Fig. 3D).

**Figure 4:**
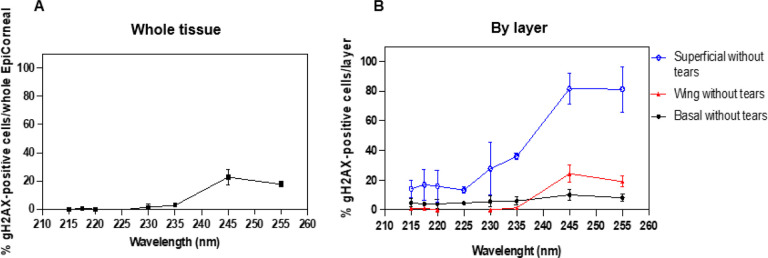
gH2AX foci formation in the whole corneal epithelium and in each layer of tissues exposed to different monochromatic UVC wavelengths. Percentage of corneal cells exhibiting gH2AX foci 24 h after exposure to 100 mJ/cm^2^ from to different individual UVC wavelengths measured in A) the whole tissue or B) in each layer of the corneal epithelium. Values represent the average ± SD of cells exhibiting gH2AX foci measured in at least six randomly selected fields of view per samples (n = 3; up to 1000 cells per sample were counted).

Next, we measured the percentage of cells positive for γH2AX foci, an established marker of radiation-induced DNA double strand breaks [34], as a function of wavelength and 24 h after exposure to 100 mJ/cm^2^ (Fig. 4). The highest percentage of cells positive for lasting γH2AX foci (approximately 20%) was measured in tissues exposed to 245 nm and 255 nm (Fig. 4A). However, for all wavelengths, γH2AX-postive cells were located mainly in the superficial layers of the corneal epithelium (Fig. 4B).

### Markers of corneal cell damage as function of UVC wavelength

**Figure 5:**
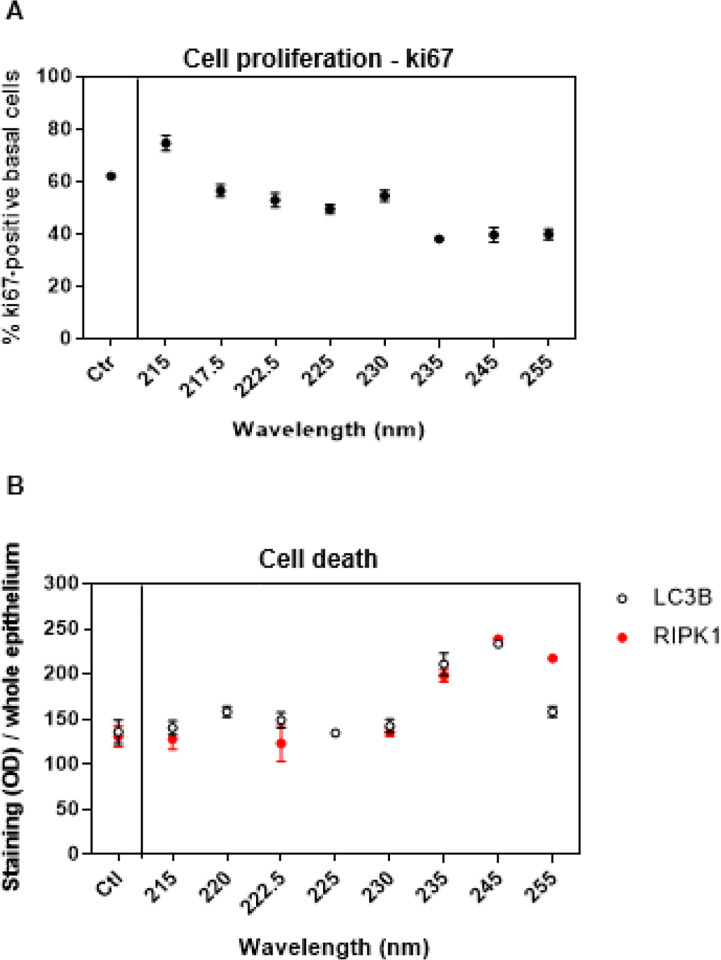
Markers of basal cell proliferation and of cell death in the whole corneal epithelium as function of UVC wavelengths. A) Percentage of basal corneal cells positive for the proliferation marker ki67 and B) immunostaining (mean gray value) of the autophagy marker LC3B and necroptosis marker RIP1 in the whole corneal epithelium. Values represent the average ± SD of cells exhibiting the markers in at least six randomly selected fields of view per samples (n =3). For ki67 between 300 and 500 basal cells per sample were counted.

Focusing again on the far-UVC range of wavelengths studied here, the absence of DNA damage in the germinative layer of the cornea (Figs. 1 and 3) does not rule out that absorption of light in the superficial cells could indirectly trigger molecular pathways leading to other types of cells and tissue damage. Therefore, we investigated the response of other biomarkers of corneal cell integrity and function 24 h after exposure to 100 mJ/cm^2^ from different individual wavelengths. We measured the proliferation marker ki67 [35] exclusively in basal cells because terminally differentiated, noncycling corneal epithelial subrabasal cells do not significantly express cell cycle markers such as ki67 [36]. We found that only in tissues exposed to 235, 245, or 250 nm less basal cells (approximately 40% on average) than controls (approximately 62%) were actively proliferating (p < 0.02) (Fig. 5A).

We then investigated the induction of different modes of cell death, including autophagy and necroptosis. Both the expression of the autophagosome marker LC3B [20] and RIP1, a necroptosis marker [21] were lower than controls only in tissues exposed to 235, 245, or 250 nm (p < < 0.001) (Fig. 5B). Notably, we did not detect apoptosis by neither expression of caspase 3 nor TUNEL assay (data not shown).

**Figure 6:**
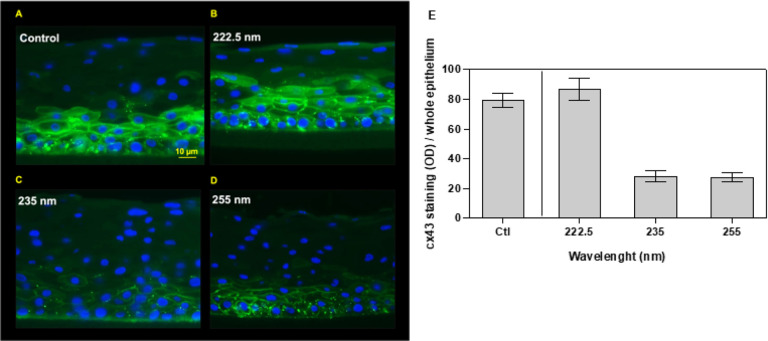
Cx43-mediated gap-junction intercellular communication in the whole corneal epithelium as function of UVC wavelengths. Representative images of tissues that 24 h earlier had been A) sham-irradiated or exposed to 100 mJ/cm^2^ from B) 222.5 nm, C) 235 nm, or D) 255 nm. Blue= 4’,6-diamidino-2-phenylindole (DAPI), green= anti-cx43 antibody conjugated to the fluorescent secondary antibody Alexa 488. E) Quantification of cx43 signal (mean gray value) as function of wavelength.

In the basal cell layer of the human corneal epithelium, cells regulate tissue homoeostasis by allowing the intercellular passage of small molecules through gap junctions whose main associated protein is connexin 43 (cx43) [22, 23, 37]. We found that cx43 expression in tissues exposed to far-UVC wavelengths like 222 nm was similar to control (Fig. 6A, 6B, and 6E); in contrast, exposure to 235 nm or higher wavelengths like 255 nm led to a disruption of the cx43-mediated intercellular communication (p < < 0.001) (Fig. 6C to 6E).

## Methods

### Monochromatic wavelength UVC exposure system

An optical system was assembled to enable monochromatic UVC exposures to 3D models of human cornea tissue. An EQ-77 Laser-Driven Light Source (Energetiq Technology, Inc., Wilmington, MA) provided a high brightness broadband output across the wavelength range of 170 nm – 2500 nm. A pair of off-axis parabolic mirrors focused the EQ-77 output into a Cornerstone 260 ¼ m monochromator (CS260-RG-2-FH-A, Newport, Irvine, CA). The monochromator was equipped with a 1201.6 g/mm plane blazed holographic reflection grating (#53-*-200H with master no. 5482, 250 nm nominal blaze wavelength, Newport) to maximize optical throughput in the UVC. Fixed slits with a slit size of 600 μm (77216, Newport) were used for all experiments. The output of the monochromator was reflected downward using an off-axis replicated parabolic mirror with an aluminum coating (50329AL, Newport) to permit the exposure of samples from above. A system warm-up time of 30 minutes was allowed for all experiments.

### Monochromatic UVC characterization and dosimetry

The monochromator spectral output was characterized using a BTS-2048UV Spectroradiometer (Gigahertz-Optik, Inc., Amesbury, MA). With a 600 μm slit width and the 1201.6 g/mm grating, the resolution of the monochromator was 1.9 nm. The measured full width at half maximum was between 2.0 nm and 2.2 nm for all peak wavelengths used in this study. The monochromatic spectral output has been previously characterized [30]. The throughput of the system was measured using an 843-R optical power meter (Newport) with a recently calibrated 818-UV/DB silicon detector (Newport). The total optical power output was measured for each wavelength examined in this work. The irradiance at the target surface was determined by dividing the optical power by the beam area at the exposure plane. The beam area was characterized by using a piece of ultraviolet sensitive film (OrthoChromic Film OC-1, Orthochrome Inc., Hillsborough, NJ) [38, 39]. The film was placed at the exposure plane and irradiated to cause a color change illustrating the total exposure area. This area was approximately an 8 mm × 10 mm ellipse, with an area of 62.8 mm^2^. Film was also used to verify the beam was fully within the detector area when measuring optical power immediately following the off-axis parabolic mirror; the beam was approximately an 8 mm × 10 mm ellipse, so it was within the 10 mm diameter detector area. The total exposure time for a given wavelength was determined by dividing the desired radiant exposure dose by the irradiance. Based on measurement uncertainties for the dimensions of the elliptical exposure area to be within ± 0.5 mm and the uncertainty of the optical power sensor of ± 4.2% for 200–219 nm and ± 2.6% for 220–349 nm, the total uncertainty of the irradiance values is estimated to be approximately ± 10%.

### UVC fixtures and dosimetry

The percentage of corneal cells showing CPD dimers in whole tissues were assessed after exposure to 50, 100 or 150 mJ/cm^2^ generated by a filtered (Ushio America, Cypress, CA) krypton-chloride (KrCl) excimer lamp (High Current Electronics Institute, Tomsk, Russia), which emits principally at 222 nm or by a low-pressure mercury lamp (Mineralight XX-15S, UVP, Upland, CA), which principally emits at 254 nm. Irradiance was measured with a calibrated Hamamatsu UV Power Meter (C9536/H9535–222, Hamamatsu Photonics K.K., Japan). For the 222 nm and the 254 nm lamps an irradiance 0.5 mW/cm2 was obtained by positioning the samples at 15 cm and 10 cm, respectively, from the lamp.

### Exposure of tissues with artificial tears

The tear film covering the surface of the cornea was simulated in these experiments. The key parameters of the tear film for these purposes were the protein concentration, which affects the absorbance and the thickness of the tear film. The tear solution for these experiments was simulated using bovine serum albumin (BSA) in Dulbecco’s phosphate buffered saline (DPBS) at a concentration of ~ 15 mg/ml [40]. While the tear film thickness is variable due to evaporation between blinking, recent measurements of the muco-aqueous layer thickness have been reported in the range of 3–5 μm [41]. This experiment used a 10 μm pathlength quartz cuvette (20/O-Q-0.01, Starna Cells, Inc., Atascadero, CA) to contain the artificial tear layer. The cuvette was filled with the BSA solution and placed in the light path prior to the exposed tissue to simulate the presence of the tear film on the cornea. The 10 μm path of BSA represents a conservative model for the tear film.

### Immunohistochemical measurement of UV-induced damage in human cornea models

We used the 3D human cornea model EpiCorneal^™^ (MatTek Corp., Ashland, MA, USA). The models consist of donated primary normal human cornea cells grown until confluence and then exposed to an air–liquid interface to stimulate tissue maturation. The 3D *in*-*vitro* reconstructed human corneal tissue model possesses similar tissue structure, barrier properties, and expression of cornea-specific markers to the *in-vivo* human cornea [6].

Samples were exposed using the monochromator to deliver 100 mJ/cm^2^ from each individual wavelength 215, 217.5, 220, 222.5, 225, 227.5, 230, 232.5, 235, 237.5, 240, 242.5, 245, 250, or 255 nm. Thirty minutes after exposure the tissues were fixed in formalin and later cut into 5-μm sections. Tissue sections were analyzed using immunohistochemistry to measure, as a function of the wavelength, induction of the two most abundant pre-mutagenic DNA lesions, CPD and 6–4PP [29]. Separate tissues were fixed in formalin twenty-four hours after exposure to selected wavelengths (215, 217.5, 220, 225, 230, 235, 245, or 255 nm). Sections of those tissues were used to measure: i) Formation of gH2AX foci – a marker of radiation-induced DNA double strand breaks [34]; ii) the percentage of proliferating corneal epithelial cells expressing the ki67 antigen [42]; iii) the expression of markers of cell death such as LC3B, a marker autophagy [20] and RIP1, a marker of necroptosis [21]; and iv) the expression of cx43, a marker of gap-junction mediated intercellular communication [22]. Sham-exposed tissues served as controls. All the biomarkers were detected with the immunohistochemical methods previously described [43] using the following antibodies: anti-CPD (1 800 in blocking buffer), mouse anti-6–4PP (1:300) (NMDND002 and NMDND002, respectively, Cosmo Bioscience USA, Carlsbad, CA, USA); anti-phospho-histone H2A.X (Ser139) (1:400) (2577, Cell Signaling Technology, Inc. Danvers, MA); all from Abcam (Cambridge, UK) were anti-ki67 (ab15580, 1:500), anti-LC3B [EPR18709] (ab192890, 1:1000), anti-RIP [EPR24883–85] (ab300617, 1:100), and anti-cx43 (ab11370–1001, 1:1000). We used Alexa 488 (1:1000, Invitrogen) as secondary antibody for the fluorescence detection of anti-cx43. All the other antibodies were detected by DAB (3, 3’-diaminobenzidine) staining. For each tissue, multiple randomly selected fields of view were analyzed across the tissues to determine either CPD, 6–4PP or γH2AX yields in the different layers of the epithelium (superficial layer, suprabasal (Wing) layer and basal layer; Fig. 1A), as well as their average over the entire epithelium. Ki67 yields were measured only in basal cells. The data represent the average ± standard deviation of cells exhibiting the damage divided by the total number of cells measured in a randomly selected fields of view. For each field of view, the total number of cells were counted as the number of nuclei positive for 4′,6-diamidino-2-phenylindole (DAPI) using the coverslip mounting medium containing DAPI (Vectashield, Burlingame, CA). Similarly, the percentage of corneal cells positive for a specific DNA damage type in each layer of the epithelium was obtained by dividing the number of positive cells in that layer by the total number of cells counted in that specific layer [30]. Tissues were examined at an Olympus IX70 microscope equipped with a Photometrics^®^ PVCAM high-resolution, high-efficiency digital camera and the Image-Pro Plus 6.0 software. For the biomarkers LC3B, RIP, and cx43, images of several fields of view of the entire epithelium were taken for each sample and analyzed with Fiji / Image J. In the case of LC3B and RIP, the images were deconvoluted using the H DAB vector and quanti ed as mean gray values. For cx43, the green-fluorescence images (*i.e*., Alexa 488-conjugated anti-cx43 antibody) were inverted and cx43 expression was quantified as mean gray values.

### Statistical analysis

DNA photodamage yields represent the average ± standard error of the mean (SEM) of keratinocytes exhibiting dimers measured in between three and 15 randomly selected fields of view per samples (two technical and two biological replicates for each sample per wavelength). Per each sample, an average of 1500 or 400 cells were counted for DNA damage or ki67 assessment, respectively; optical density (mean gray value) to assess expression of LC3B, RIP1, and cx43 was measured in six to 15 fields of view (three technical and one biological replicates for each sample per wavelength). For the samples exposed to light generated by the 222- or 254-nm fixtures, values represent the average ± SD of cells exhibiting dimers measured in three to eight randomly selected fields of view per samples (n = 2, an average of approximately 600 cells per sample were counted). Comparisons of mean values between treatment groups and controls were performed using the Student’s t test. For comparison of the percentage of CPD-positive cells in tissues exposed with or without simulated tears in the whole epithelium or within its different layers, logistic regression was used to model the CPD-positive cells divided by the total scored cells proportion. Fisher’s exact test was used to compare the yield of CPD-positive cells per cell scored in each of the three corneal layers, with corresponding zero-dose controls. Raw p values and p values were adjusted by the Bonferroni procedure for multiple comparisons.

## Discussion

Using a monochromatic source, we studied the biological response of a 3D model of the human cornea exposed to 100 mJ/cm^2^ generated by individual UVC wavelengths. We conducted the first set of experiments with tissues exposed with or without simulated tears to test the hypothesis that the proteins present in the tears would absorb the incident light and reduce the induction of DNA photodamage in corneas. Our results showed that exposure to UVC light in the presence of tears reduced the induction of DNA dimers to a degree dependent on the specific wavelength and tissue layer (Fig. 1). In the cells of the basal layer of the cornea, which are indispensable to initiate tissue regeneration, there was no significant increase in measured DNA damage at wavelengths below 240 nm (with tears) or 237.5 nm (without tears).

It is important to note that our model of human tears consisted of a simple 10 μm layer of ~ 15 mg/ml of BSA, which is a 68 kDa globular non-glycoprotein made of 583 amino acid residues of both carboxyl and amino groups. In reality, more than 1500 proteins have been identified in the human tear film [14], which consists of an outer lipid layer with intercalated proteins, a middle aqueous phase containing ~ 11 mg/ml proteins [40], small molecule metabolites, electrolytes, and gel-forming mucins, and an inner glycocalyx layer rich in transmembrane glucoproteins and mucins [44]. Modeling the human tear film is challenging because its composition and thickness vary among individuals, being as low as approximately 3 μm to up to 40 μm [14, 41, 45]. From the perspective of the incident light, due to constant eye blinking, the three-phase tear film is also highly dynamic making the distribution of proteins and lipids overlaying the eye not uniform.

The second set of experiments was conducted with samples exposed to monochromatic UVC light without tears. We measured induction of DNA dimers 30 minutes after exposure and found that all the wavelengths studied induced CDP and 6 – 4 PP; notably, the wavelengths in the far-UVC range (215 nm to 235 nm) induced dimers only in the uppermost superficial layers of the epithelium (Fig. 3).

Exposure to UVC light induces small amounts of DNA double strand breaks not by direct interaction with the DNA but rather because of errors during the replication of unrepaired lesions such as CPD [46–48]. When the DNA replication machinery encounters a replication-blocking lesion, DNA polymerase stalls at the replication fork and forms a Y-shaped DNA structure that is recognized by endonucleases which, in turn, generate a nick in the template strand resulting in induction of a double strand break near the replication-blocking lesion [49]. In principle, due to their higher photon energy, shorter wavelengths like far-UVC could induce more double strand breaks, which are typically more difficult to repair, than the longer wavelengths tested in this study [11]. We found that this was not the case, in fact 24 h after exposure, far-UVC wavelengths did not cause lasting DNA double strand breaks (γH2AX foci), which in contrast were still detected in cells of tissues exposed to 245 nm and 255 nm, although they were located mainly in the uppermost layers of the epithelium (Fig. 4). Like in other stratified epithelia, cells in the upmost layers of the cornea are postmitotic and are destined to be sloughed off during normal replicative turnover [32]. Thus, concerns regarding short- and long-term cornea safety after exposure to UVC light relates to DNA damage induced in cells in the basal layer, which contains the progenitor cells [50]. Nonetheless, one cannot exclude that the DNA damage induced in the superficial layers of the tissue could trigger damaging molecular pathways propagating downstream to cells in the deeper layers of the corneal epithelium. The ensuing detrimental biological effects may vary significantly by wavelength.

Biomarkers such as basal cell proliferation and cell-to-cell intercellular communication can be directly associated to tissue functions. Corneal gap junctions mediate the intercellular transfer of ions and low molecular weight metabolites in basal epithelium and stroma and thus are vital for corneal tissue differentiation and overall tissue homeostasis. One of the main proteins involved in corneal gap junctions is cx43, which is widely expressed throughout the layers of the corneal epithelium, with the exception of the most superficial corneal cells [22]. We showed that 24 h after exposure to 100 mJ/cm^2^ the wavelengths in the far-UVC region, unlike longer wavelengths, did not halt proliferation of the germinative cells (ki67) (Fig. 5A) nor impair cell-to-cell intercellular communication (cx43) (Fig. 6).

Cell death can be considered as a link between cellular damage and the response of the whole tissue to UVC exposure; while it can be beneficial for the tissue to remove a few damaged cell, excessive cell death may cause the failure of the whole tissue. Programmed cell death is categorized as nonin ammatory (*e.g*., apoptosis) or in ammatory (*e.g*., necroptosis) [51]. While apoptosis and autophagy remove damaged cells and proteins and protects the tissue by reducing the production of in ammatory cytokines, necroptosis is considered a programmed form of necrosis, which is characterized by loss of cell membrane integrity and the release of inflammatory molecules into the extracellular milieu [51, 52]. We found that for all the wavelengths studied, none triggered apoptosis (data not shown). Only longer wavelengths (i.e., 235 nm, 245 nm, or 250 nm) elicited an increase in the expression of the autophagosome marker LC3B [20] and the necroptosis marker RIP1 (Fig. 5B). It has been established that the type of insult / stimuli (*e.g*., abrasion, UV, infection, etc.) preferentially triggers one cell death pathway rather than another (*e.g*., apoptosis, necrosis, autophagy, etc.) [53], and our results may reflect just that. While apoptosis is typically initiated by programmed tissue turnover or by microenvironmental perturbations like growth factor deprivation or reactive oxygen species overload, necroptosis and autophagy are mainly triggered by perturbations of cellular homeostasis as an adaptative response to stress. In corneas, it has been speculated that the cellular disintegration phase in necroptosis serves to attract immune cells and to initiate regeneration processes [53].

In summary, our results indicate that among the wavelengths studied, 100 mJ/cm^2^ from far-UVC wavelengths did not induce detrimental responses in the critical basal layer of the epithelium of a 3D model of the human cornea, whereas longer wavelengths did, to an extent dependent on the biological endpoint. These conclusions are similar to what we have previously observed in a model of human skin [30]. Collectively, our studies support the notion that when used within regulatory safety limits, far-UVC sources can be considered safe to be used in occupied locations where direct exposure of human skin and eyes is possible [13, 54–57], such as in applications designed to reduce the risk of transmission of airborne diseases in occupied locations.

This study has some limitations. A more realistic model of the human tear film should be developed to con rm the extent to which tears protect the human cornea from UVC light. More representative human tear film models would allow studying the response to UV exposure of sensitive individuals such as people suffering from dry eyes and other disorders associated to tear film dysfunctions [58].

The biological response of a model of a human tissue partially represents the response of the tissue within the organism. Cornea models that include resident immune cells and sensory nerves would allow to investigate neuro–immune interactions [59] that, upon exposure to UV light, may result in ocular surface discomfort [60]. Nevertheless, the model used in this study structurally and functionally behaves like native human corneas [6] and represents a useful tool to compare the response of different biomarkers as a function of UVC, which can inform the design of other experiments and contribute insights to interpret animal and human studies.

## Figures and Tables

**Table 1:** Wavelengths (nm) above which measured corneal DNA damage was significantly greater than the control (zero-dose) values.

	Corneal Layer	Wavelength (nm)
	Basal	240
**With tears**	Intermediate	230
	Superficial	215
	Basal	237.5
**Without tears**	Intermediate	232.5
	Superficial	215

## Data Availability

Raw data can be accessed from the Open Science Framework repository, DOI 10.17605/OSF.IO/QAV96.
